# Host-targeted oral avian vaccine virus demonstrates broad antiviral activity and safety in patients

**DOI:** 10.3389/fimmu.2026.1760109

**Published:** 2026-02-25

**Authors:** Tibor Bakacs, Konstantin Chumakov

**Affiliations:** 1 Hungarian Research Network (HUN-REN) Alfréd Rényi Mathematical Research Institute, Budapest, Hungary; 2 HepC Therapeutics Inc., Budapest, Hungary; 3 George Washington University, Washington DC, United States

**Keywords:** Broad-spectrum antiviral, Double-stranded RNA (dsRNA), host-directed therapy, infectious bursal disease virus (IBDV), interferon response

## Abstract

The absence of an immediately deployable, broad-spectrum antiviral remains a critical vulnerability in global pandemic preparedness. Host-directed agents that activate innate immunity offer a pathogen-agnostic strategy, yet no such therapy is currently stockpiled or authorized for emergency use. Infectious Bursal Disease Virus (IBDV)—a non-replicating avian dsRNA vaccine virus with a 60-year safety record in poultry—induces robust interferon responses in mammals and has been administered orally in marmosets and more than 50 human patients with hepatitis A, B, C, SARS-CoV-2, and herpes zoster infections. These observations include a randomized phase II trial in 84 acute HBV/HCV patients. Although the evidence base is limited, the consistency of clinical responses and absence of serious safety signals justify renewed scientific examination. This review synthesizes the mechanistic rationale, comparative advantages over synthetic Pattern Recognition Receptor (PRR) agonists, clinical observations, One Health implications, and regulatory precedents relevant to evaluating IBDV as a temporary, compassionate-use antiviral during pandemics while the reverse-engineered human candidate (IBDV-R903/78) progresses through formal development. The goal is not to endorse clinical deployment, but to initiate a rigorous, multidisciplinary debate on whether an established veterinary dsRNA vaccine virus could serve as an off-the-shelf host-directed live viral adjuvant therapy in future public health emergencies.

## 1 Introduction

“There is about a 50% chance that a new pandemic causing 25 million or more deaths will occur between now and 2050” warned Jamison et al. (1). Among many lessons from COVID-19 pandemic is the realization of our vulnerability and unpreparedness to the emerging threats. The consensus is that the next pandemic is not a matter of if, but rather when. However, the exact pathogen that will cause it cannot be predicted with any level of certainty, making the development of specific countermeasures a major challenge. The development of broad prophylactic and therapeutic strategies that would not rely on the development of pathogen-specific vaccines but rather on the activation of host innate immune mechanisms could provide a solution. Several such strategies were proposed in the past, however so far none of them came to fruition, mostly because they were based on infectious agents with uncertain human safety. Here we propose an approach based on a harmless double-stranded RNA animal virus that cannot infect humans but induces broadly protective interferon response upon oral administration.

Although promising research is underway—including efforts to target common viral features like surface glycans—a truly broad-spectrum antiviral that works across multiple viral families is still in development. Programs like NAVIPP and the INTREPID Alliance are accelerating the discovery and clinical readiness of broad-spectrum antivirals. But as of now, these candidates are not yet stockpiled or approved for emergency use. This lack of a deployable broad-spectrum antiviral means that in the early stages of a new outbreak, the world must rely on containment measures (e.g. lockdowns, quarantines), supportive care, rapid development of virus-specific treatments and vaccines.

The International Committee on Taxonomy of Viruses (ICTV) has classified over 6000 viruses, with hundreds known to infect humans and their number is growing (2). The inconvenient truth, however, is that antiviral drugs have been formally approved for the treatment of only 9 human infectious diseases (3). The average development cost of a new drug is $2.6 billion and requires 10 to 15 years from discovery to regulatory approval. Clearly, the gap between “bugs and drugs” cannot be closed by conventional drug development. The traditional model of antiviral development is not only slow and expensive, but also ill-suited for rapid pandemic response due to its virus-specific nature (4).

Despite decades of research and billions in investment, antiviral therapeutics remain narrow in scope, slow to develop, and susceptible to resistance. In contrast to antibiotics, which can target a broad range of bacterial pathogens, most antiviral drugs are designed to inhibit specific viral proteins or replication mechanisms. While this targeted approach can be effective, it means that each drug is typically tailored to a single virus or viral family, rendering it ineffective against newly emerging or mutating viral threats.

The process of identifying viral targets, screening compounds, and obtaining regulatory approval can take years—time that is often unavailable during rapidly evolving outbreaks. Furthermore, viruses mutate quickly, and even successful antivirals, such as those used against HIV or influenza, are continually challenged by viral evolution, which can lead to resistance and reduced efficacy.

In short, the antiviral landscape is fragmented and reactive. It lacks a universal, broad-spectrum solution that can be deployed quickly and effectively across a range of viral threats. This gap is especially dangerous in the context of zoonotic diseases, where novel viruses emerge unpredictably from animal reservoirs and spread rapidly through human populations (5).

To address this gap, we proposed a fundamental shift in the approach to combating pathogenic viruses (6). The infectious bursal disease virus (IBDV) antiviral drug candidate, developed based on the innovative concept of superinfection therapy, has the potential to redefine the landscape of antiviral treatment.

## 2 Fighting fire with fire: controlling unrelated infections by live viral adjuvant therapy

The idea of using viral interference induced by live viral vaccines to protect against unrelated infections was proposed many years ago (7). Recently the mechanism of broadly specific protection started to be uncovered and led to the formulation of trained innate immunity concept (8). The use of existing live viral vaccines such as oral polio vaccine (OPV) (9) was explored during the recent COVID19 pandemic (10), but did not get widely accepted primarily because of the concerns about potential spread of vaccine-derived polioviruses. Therefore, finding a suitable agent to induce broadly specific protection against a range of viral infections is an important priority.

By harnessing an avian virus that is harmless to humans, IBDV represents a radically new approach: a potential “antibiotic for viruses” (11). The double-stranded RNA (dsRNA) of IBDV exhibits species-specific effects: in its natural chicken hosts, it suppresses interferon (IFN) responses, whereas in species that do not support its replication, such as mammals, it acts as a potent inducer of IFN responses (12–14). The antiviral effect of IBDV arises from PRR activation rather than competition between replicating viruses. The term *live viral adjuvant therapy* more accurately describes this mechanism.

This property makes the long dsRNA of IBDV a valuable tool for priming antiviral defenses in mammals. Notably, IBDV has been used in billions of poultry vaccinations over more than 60 years without any cases of zoonotic transmission (Merck Veterinary Manual).

The IBDV drug candidate offers, therefore, a fundamentally different approach—one that could bypass the limitations of target-specific design and resistance-prone mechanisms. IBDV functions essentially as a viral Toll Like Receptor/Pattern Recognition Receptor (TLR/PRR) agonist without bona fide infection. In such a way, IBDV functions as a live viral adjuvant therapy, which holds the potential to act as a broad-spectrum antiviral, disrupting pathogenic viruses regardless of their genetic makeup or origin.

IBDV-R903/78 is a live, attenuated dsRNA virus that engages the innate sensing network in a *physiological*, spatially and temporally regulated manner. In mice, a single dose induces a coordinated interferon and ISG program—including Irf7, Ifi204, Zbp1, Cxcl10, Tlr3, Tlr9 and others—consistent with crosstalk between PRRs, TLRs and the cGAS–STING axis, without excessive type I IFN or pro-inflammatory cytokine release (15). In patients, repeated oral IBDV administration has shown antiviral efficacy against HAV, HBV, HCV, SARS-CoV-2 and HZV, with long-term use even in decompensated liver disease, and without cytokine storm or serious safety signals, despite high viremia and prolonged “artificial viremia”. This clinical experience—across multiple viral families and indications—demonstrates that IBDV can safely “tune” innate immunity rather than overdrive it. IBDV offers a naturally evolved dsRNA structure with decades of real-world safety data in animals and observational safety data in humans.

By contrast, synthetic TLR7/8/9 agonists (vesatolimod/GS-9620, RO7020531, JNJ-4964, selgantolimod/GS-9688, AIC649) are pharmacologically potent but their safety window is still being defined. In early and mid-stage trials, these agents consistently show dose-limiting, systemic immune activation—flu-like symptoms, pyrexia, headache, gastrointestinal events, cytopenias, and transaminase elevations—reflecting off-target or excessive on-target stimulation of TLR pathways. For example, selgantolimod (GS-9688, TLR8 agonist) was “safe and well tolerated” only within a narrow dose range, with higher doses limited by systemic adverse events and modest antiviral efficacy in chronic hepatitis B (CHB) infection (16). Similarly, RO7020531 (TLR7 agonist) in healthy volunteers and CHB patients required careful dose titration because of systemic reactogenicity and immune-activation–related AEs (17). These profiles underscore that further work is needed to fully characterize long-term safety, optimal dosing, and combination strategies for TLR agonists (18) (19).

## 3 IBDV sits at the intersection of veterinary and human medicine as a prototypical One Health antiviral

Major animal disease outbreaks are currently managed largely through mass culling, movement restrictions, and trade bans. Since 2000, over 350 major animal disease outbreaks have been reported globally, with more than a quarter due to avian influenza and most of the remainder caused by a small set of highly contagious infections such as foot-and-mouth disease, classical swine fever, Newcastle disease, and African swine fever. These events have profound economic and social consequences, including loss of animal capital, disruption of supply chains, and long-term impacts on rural livelihoods and food security. A host-targeted, orally administered, broadly antiviral agent such as IBDV could, in principle, offer an alternative to blanket culling in some scenarios by rapidly inducing antiviral defenses in at-risk herds or flocks.

The One Health paradigm encourages integrated approaches to human, animal, and environmental health. Increasingly, immunological insights from veterinary medicine inform human therapeutics. IBDV, as a non-replicating dsRNA virus with potent interferon-inducing properties, fits squarely within this translational logic. Its oral delivery format, long safety record in poultry, and lack of zoonotic transmission provide a strong rationale for considering its use not only in humans but also as a tool to mitigate animal pandemics themselves.

Plausible applications would be high-impact transboundary animal diseases. In swine, classical swine fever and African swine fever have led to the destruction of hundreds of millions of pigs globally, with cascading effects on feed markets, meat prices, and rural economies (20). In ruminants, foot-and-mouth disease outbreaks have historically triggered large-scale culling and trade bans; even modest reductions in viral shedding and clinical severity could translate into substantial savings by limiting the geographic spread and duration of trade restrictions. The economic stakes are enormous. Emergency animal diseases routinely generate direct and indirect costs in the range of hundreds of millions to several billions of US dollars per major outbreak, once production losses, culling, disposal, surveillance, and trade impacts are accounted for (21).

If future controlled studies in livestock confirm that oral IBDV administration can safely induce a protective antiviral state against diverse viral pathogens, it could become a practical alternative or adjunct to mass culling during animal pandemics. Even partial replacement of culling with IBDV-based “antiviral flock treatment” could prevent the destruction of tens of millions of animals per outbreak and avert economic losses in the order of billions of dollars, while simultaneously reducing the risk of zoonotic spillover and aligning with ethical and societal expectations for more humane disease control.

Thus, IBDV represents a rare candidate with *dual-use potential* for human and veterinary pandemics. To protect humans from viral pandemics and to protect animal populations from devastating epizootics—underscores the need for coordinated research and policy frameworks that evaluate IBDV not only as a human therapeutic, but also as a strategic tool to replace or reduce culling in animal pandemics and thereby mitigate their massive economic and societal costs.

## 4 Clinical studies with avian IBDV vaccine demonstrated safety and efficacy in marmoset monkeys and patients

Experimental IBDV therapy has been validated in marmoset monkeys and patients against hepatitis A, B, C viruses, SARS-CoV-2 virus and herpes zoster virus (**Table 1**). Although limited, the human experience with IBDV includes both controlled and uncontrolled observations.

**Table 1:** Human and Non-Human Primate Experience with Oral IBDV.

Type of virus	Number of subjects	Study type	References
Hepatitis A virus (HAV)	20 marmoset monkeys	Controlled study	(22)
Hepatitis B virus (HBV)	20 acute and 2 decompensated patients	Randomized phase II + case series	(23, 24)
Hepatitis C virus (HCV)	22 acute and 2 decompensated patients	Randomized phase II + case series	(23, 24)
COVID-19 disease (SARS-CoV-2)	5 acute patients	Case series	(25)
Shingles (herpes zoster virus; HZO)	1 severe patient	Case report	(26)

COVID-19, coronavirus disease 2019; SARS-CoV-2, severe acute respiratory syndrome coronavirus 2; IBDV, infectious bursal disease virus. Reproduced with permission from Bakacs, T., Volker, S., and Kovesdi, I. (2022d). An Orally Administered Nonpathogenic Attenuated Vaccine Virus Can Be Used to Control SARSCoV-2 Infection: A Complementary Plan B to COVID-19 Vaccination. Cureus 14, e28467. 10.7759/cureus.28467.

All published human studies to date on more than 50 patients have used the conventionally produced veterinary IBDV vaccine. Originally the MTH68/B strain of IBDV has been used in patients, which is a live attenuated IBDV produced under GMP condition by Phylaxia Veterinary Biologicals Co. (now part of CEVA Phylaxia). When production of MTH68/B for human use has been terminated, the commercially available CEVAC ^®^ GUMBO L vaccine was administered in patients. IBDV demonstrated safety and potential efficacy against five unrelated viral families including hepatitis A, B, C viruses, SARS-CoV-2, and herpes zoster virus (25).

The IBDV dataset is small, but unusually consistent: rapid symptom improvement, absence of serious adverse events, tolerability even in severe disease, activity across multiple viral families. Consistency across indications is scientifically meaningful even when sample sizes are modest.

### 4.1 A controlled phase II study in 84 patients with acute HBV or HCV infection

This controlled phase II study was conducted in 1998. Age, however, alone does not invalidate a well-conducted trial—especially when the biological mechanism (dsRNA-induced innate immunity) is timeless. The randomized phase II trial is therefore not obsolete. The study remains methodologically sound: prospective, controlled, 84 patients, clinically relevant endpoints, clear differences between groups.

Briefly, the IBDV vaccine virus (MTH68/B) was tested in 84 patients of both sexes (ages 14-70) with either a diagnosis of acute B (43 patients) or acute C (41 patients) viral hepatitis (23). Clinical and laboratory investigations were performed before treatment, weekly for one month and once every month for 6 months consisting of physical examination, detection of viral markers, liver function tests. Liver biopsy was performed in case of suspicion of chronic hepatitis. Consistently with the literature, more patients progressed into chronic hepatitis in the HCV than in the HBV group. Importantly, a significant difference between the IBDV treated and control groups was observed. Nine percent of the HCV and none of the HBV patients progressed into chronic disease, whereas 13% and 26% respectively of the controls did. Prior to complete recovery 9% of HBV and 79% of HCV control patients, but only 5% and 32% of the IBDV treated patients relapsed. Late remissions (requiring more than 6 months) were recorded significantly more frequently with conventional treatment in both HBV and HCV hepatitis groups (17% and 42% respectively in controls but 0% and only 14% with IBDV treatment). The duration of the first icteric phase was also shortened with the IBDV treatment (by 20% in the HBV and 40% in the HCV groups). In addition, remission within one month of treatment was registered more often in the virus treated groups (both 50% respectively) than in the control groups (26% and 21%). No serious adverse events (SAEs) related to IBDV treatment were noted. With the benefit of hindsight, the dose and duration of the IBDV treatment in this study was suboptimal.

### 4.2 Case studies can generate hypothesis with limited numbers of patients

Severe decompensated HBV or HCV cirrhosis with life-threatening symptoms constitutes a medical emergency, requires immediate intervention and urgent evaluation for liver transplantation. IBDV superinfection therapy was however effective in several such hepatitis patients in outpatient settings (24, 27). These patients demonstrated striking clinical improvement without significant side effects when large doses of the viral preparation were administered continuously over an extended period to maintain ‘artificial IBDV viremia’ because of its inability to replicate in mammals (28). One of the patients (a 67-year-old female with an HCV infection) was confined to bed, unable to support herself due to a severe malaise. In the second patient (57-year-old female with HBV infection), a portal hypertension developed, suggesting a very poor prognosis. In the third patient (a 24-year-old female with HBV infection) progressive jaundice, generalized edema and hepatic encephalopathy occurred and a request for liver transplantation was refused because of the high-level viremia. The fourth decompensated chronic HCV patient had a 20-year-history of the disease (29). The patient went into chronic-active hepatitis state full time and became so overwhelmed with chronic fatigue syndrome (CFS), muscle and bone pain that he was unable to walk for months. The patient was in or close to the bed for the following six years. He had high viral loads (above 5 million HCV RNA copies/ml) and high levels of liver enzymes (up to 1,600 U/L alanine aminotransferase, ALT; and 800 U/L aspartate aminotransferase, AST). Fevers and nausea were prevalent and cognitive reasoning was seriously impaired. Liver (needle) biopsy revealed chronic hepatitis with established bridging fibrosis, mild piecemeal necrosis and macrovesicular steatosis, consistent with HCV hepatitis. He received disabled status. Repeated IBDV therapy, however, proved effective enabling him to work again.

Importantly, IBDV treatment did not induce excessive release of pro-inflammatory cytokines, even in chronic HBV or HCV patients with decompensated liver disease and high-level viremia, a condition typically associated with cytokine storms (15, 30).

Severe Herpes Zoster Ophthalmicus (HZO) with orbital edema is a critical ophthalmologic emergency (31). Medical guidelines emphasize that immediate intervention is required to prevent irreversible vision loss and life-threatening systemic complications. It was, therefore, unexpected that the immunostimulatory effect of IBDV was notably demonstrated in a severe case of herpes zoster ophthalmicus, where rapid clinical improvement was observed following treatment (see figure 2 in (26)). The HZO autobiographical case report (26) of the first author of this review was not designed as an N-of-1 trial (32). Notwithstanding, with the benefit of hindsight, it would have probably suited such trial. Combination of acyclovir (ACV) and IBDV therapy had a quick onset and offset with a short treatment period. This makes it easy to measure the outcomes of interest. Simple visual inspection produced accurate conclusions to determine the results. Although anecdotal, the case has been widely read (>17,000 views), illustrating scientific interest. Based on this case report, we generated a hypothesis that could provide guidance for HZ patients. We predicted that administration of the IFN-inducing IBDV would leverage the activity of ACV by bolstering of innate antiviral immunity of the facial HZ and HZO patients. To confirm the previously demonstrated safety of IBDV-R903/78 in humans, we proposed a small randomized phase I/II trial in elderly subjects with facial HZ (33).

## 5 A reverse-engineered artificial virus (IBDV-R903/78) was developed specifically for human use

Cross-species transmission of viruses is strongly constrained by the evolutionary distance between donor and recipient hosts (34). Numerous comparative studies demonstrate that host phylogeny is a major determinant of whether a virus can establish productive infection in a new species, because viral entry, replication, and immune evasion depend on host-specific molecular compatibilities. Birds and mammals diverged hundreds of millions of years ago, and this deep evolutionary separation is reflected in substantial differences in their cellular receptors, innate immune pathways, and intracellular replication environments.

For avian influenza viruses, these host barriers are particularly pronounced. Avian influenza polymerase complexes function inefficiently in mammalian cells and typically require multiple adaptive mutations to replicate productively. Experimental evolution studies show that adaptation to mammals involves the stepwise acquisition of several mutations affecting polymerase activity, receptor binding, and host-range determinants. Although the exact number of mutations required cannot be predicted *a priori*, the available evidence indicates that substantial genetic change is needed for an avian virus to become fully competent in humans.

Swine play an important ecological role in this process because they are susceptible to both avian and human influenza viruses (35, 36). Their respiratory tract expresses both avian-type (α2,3-linked) and human-type (α2,6-linked) sialic acid receptors, enabling co-infection and facilitating reassortment or adaptation. This makes swine an important intermediate host in which avian influenza viruses can acquire mammalian-adapted traits before infecting humans.

Fortunately, IBDV does not have such a natural genetic engineering laboratory. Consistent with this, no zoonosis cases have ever been reported over the past 60 years during IBDV mass vaccination programs in poultry. Notwithstanding, addressing regulatory concerns about mutation risk and enabling rapid scale-up, an artificial virus (IBDV-R903/78 strain) was created by reverse engineering, which is genetically identical to the veterinary IBDV vaccine. The safety of the new IBDV R903/78 viral drug candidate was tested in animal studies (28) and in 15 stage IV cancer patients (including colon, stomach, pancreatic, breast, ovarian, prostate, bladder, head and neck cancers, glioblastoma, melanoma, myeloma), who were resistant to all conventional therapy. Even very high doses of R903/78 virus (1000-fold higher than the lowest *in vivo* effective dose) were associated with only mild flu-like adverse effects (37). During the COVID-19 pandemic, both the German Paul Ehrlich Institute (PEI) and the USA NIH ACTIV Agent Prioritization Team reviewed our data and acknowledged the merit of IBDV-R903/78 as a potential treatment for SARS-CoV-2. Genetic equivalence provides a scientific basis for compassionate use of the veterinary version while human trials are underway.

Regulatory pathways for Emergency Use Authorization (EUA) and Compassionate Use (CU) programs exist in both FDA and EMA frameworks to allow deployment of unapproved therapies during public health crises. When patients are dying without treatment, withholding a safe, potentially life-saving therapy due to bureaucratic delay is ethically indefensible.

IBDV-R903/78 can be stockpiled and deployed immediately in the event of an outbreak. As a host-targeted therapy, it bypasses viral escape mechanisms and can be used in conjunction with vaccines and antivirals, either as an adjuvant or as a first-line defense. Its oral administration and low cost facilitate rapid and widespread deployment. Given the increasing risk of zoonotic diseases associated with large-scale animal use (5), the need for such a broad-spectrum antiviral solution is more urgent than ever.

## 6 Considering provisional emergency deployment of veterinary IBDV vaccine to mitigate pandemic mortality

Pandemics pose an existential threat to global health and economic stability, as evidenced by the COVID-19 pandemic’s devastating toll on lives and economies (WHO, 2025 (38–41)). Despite the availability of a safe, attenuated veterinary IBDV vaccine with demonstrated antiviral properties, regulatory inertia continues to delay its deployment for human use. This brief, therefore, advocates for an immediate discussion about the compassionate use of the veterinary IBDV vaccine as a broad-spectrum antiviral during pandemics, leveraging existing emergency frameworks to save lives and mitigate economic collapse.

Dr. Michael Osterholm’s warning in *The Big One* underscores the urgency: “What will it take to convince us that the greatest threat we face to our national security and the way of life is an invasion of deadly microbes? We may find ourselves needing that planning far sooner than we expect. The time to start is now.”

An influenza pandemic is inevitable. It could infect 30% of the global population and cause over 200 million deaths in the first wave. We are a single mutation away from a human H5N1 pandemic strain (42). The COVID-19 pandemic revealed a catastrophic mortality and cost (over US$12 trillion) of delayed therapeutic interventions (43). The attenuated veterinary IBDV vaccine—used safely in billions of poultry for over six decades with no zoonotic transmission—offers a unique opportunity to deploy a pathogen-agnostic antiviral tool immediately.

### 6.1 Scientific rationale

The IBDV vaccine is a potent inducer of interferon responses (15) and demonstrated broad antiviral effects in patients. Administration of IBDV in marmoset monkeys and patients demonstrated safety and potential efficacy against five unrelated viral families, including hepatitis A, B, C, SARS-CoV-2, and herpes zoster virus. No patients experienced significant adverse outcomes (23, 24). In all the published human studies a conventionally produced veterinary IBDV vaccine was used in more than 50 patients. The veterinary strain is genetically identical to the reverse engineered human IBDV-R903/78 drug candidate, which remains years from approval.

### 6.2 Strategic advantages

Veterinary IBDV vaccines are manufactured at scale, stockpiled, globally available, inexpensive, and easy to distribute, enabling rapid deployment even in low-resource settings ( (44); Vector Vaccines Help Solve Global Infectious Bursal Disease Virus Challenges^1^; Infectious Bursal Disease Virus Variants: A Challenge for Commercial Vaccines?^2^; Infectious Bursal Disease Virus^3^). Unlike virus-specific antivirals or vaccines, IBDV can be deployed immediately without waiting for pathogen identification. The One Health Alignment of WHO supports integrated approach to human and animal health. IBDV could potentially be effective even in the early phase before pathogen identification, which is critical in the first wave of outbreaks. There are regulatory precedents such as Emergency Use Authorization (EUA) and Compassionate Use (CU) pathways, which exist for such scenarios.

Many transformative therapeutic concepts—CAR-T cells, checkpoint inhibitors, fecal microbiota transplantation, mRNA vaccines—originated from single cases or small series. The purpose of this review is to evaluate plausibility, not to prove efficacy.

## 7 Regulatory precedents for veterinary-to-human drug repurposing

The proposal to deploy IBDV as a post-infection therapeutic in pandemic emergencies may appear unconventional. However, there are well-documented precedents where veterinary drugs have been authorized or repurposed for human use, particularly under urgent or compassionate conditions. These cases demonstrate regulatory flexibility when safety, biological plausibility, and public health urgency align.

### 7.1 Ivermectin: From livestock to global health

Ivermectin was originally developed for parasitic infections in livestock and companion animals. Its potent antiparasitic activity and favorable safety profile led to its approval for human use in treating onchocerciasis and strongyloidiasis (45). The World Health Organization (WHO) has endorsed ivermectin in mass drug administration campaigns, and it remains a cornerstone of global health interventions. Its trajectory from veterinary to human medicine exemplifies the translational potential of animal therapeutics.

### 7.2 Streptomycin and aminoglycosides

Streptomycin, first used in veterinary contexts, became a foundational antibiotic in human tuberculosis treatment (46). Similarly, aminoglycosides such as gentamicin and tobramycin—initially characterized in animal models—are now standard in treating serious bacterial infections in humans. These examples reflect the historical permeability between veterinary and human pharmacology, particularly in antimicrobial development.

### 7.3 Emergency use and compassionate frameworks

Regulatory agencies such as the U.S. Food and Drug Administration (FDA), the European Medicines Agency (EMA), and Health Canada have established pathways for emergency or compassionate use of unapproved drugs. Under these frameworks, agents with established safety profiles—even if not formally approved for human use—may be deployed in life-threatening situations when no alternatives exist. The FDA’s Expanded Access Program and the EMA’s Compassionate Use provisions explicitly allow such flexibility, particularly during public health emergencies.

## 8 Limitations and future directions

The clinical trial experience is insufficient to endorse the post-infection live IBDV adjuvant therapeutical strategy as a general alternative CU/EUA option. The safety in large populations is unknown, optimal dosing, duration, and biomarkers require definition. To this end, randomized controlled Phase I/II trials in HBV and HZV patients have been proposed and are necessary (47) (33). Such randomized controlled studies should confirm or refute our proposal that the IBDV vaccine virus could be used until the reverse engineered IBDV R903/78 drug candidate will be registered as a broad-spectrum antiviral HDT.

## 9 Conclusion

IBDV is a unique antiviral candidate: a non-replicating avian dsRNA virus with a long safety record, broad interferon-inducing capacity, oral administration, low cost, and potential dual-use benefits for human and animal health. While current evidence is insufficient for clinical endorsement, it is sufficient—and unusually compelling—to justify a rigorous scientific debate about whether veterinary IBDV could serve as an off-the-shelf, host-directed antiviral during future pandemics. The question is not whether IBDV is ready for deployment, but whether we can afford to ignore a safe, scalable, pathogen-agnostic antiviral candidate in an era of accelerating viral threats.

“*The tragedy is not our inability to prevent the inevitable or to do the impossible; the tragedy is when a person, a group, or a society fails to achieve the possible*.”

—Dr. Richard Klausner (Scientist and former Director of the National Cancer Institute of the United States)

## Footnotes

1 https://agriinsightpublications.com/article/vector-vaccine-addresses-evolving-velogenic-newcastle-disease-strains-3/.

2 https://avinews.com/en/infectious-bursal-disease-virus-variants-a-challenge-for-commercial-vaccines/.

3 https://www.pirbright.ac.uk/our-science/research-groups/infectious-bursal-disease-virus.
